# Supplementary material for: Atomic models of the Toxoplasma cell invasion machinery
Source: Nat Struct Mol Biol. 2025 Dec 9;33(1):157–70. doi: 10.1038/s41594-025-01728-w (PMC12819142; doi:10.1038/s41594-025-01728-w)
Supplement: Supplementary file 1 — Reporting Summary [file 41594_2025_1728_MOESM1_ESM.pdf]

nature portfolio

Corresponding author(s): L. David Sibley, Rui Zhang

Last updated by author(s): Nov 7, 2025

Reporting Summary

Nature Portfolio wishes to improve the reproducibility of the work that we publish. This form provides structure for consistency and transparency in reporting. For further information on Nature Portfolio policies, see our Editorial Policies and the Editorial Policy Checklist.

Statistics

For all statistical analyses, confirm that the following items are present in the figure legend, table legend, main text, or Methods section.

n/a	Confirmed	
☐	☒	The exact sample size (*n*) for each experimental group/condition, given as a discrete number and unit of measurement
☐	☒	A statement on whether measurements were taken from distinct samples or whether the same sample was measured repeatedly
☐	☒	The statistical test(s) used AND whether they are one- or two-sided<br>*Only common tests should be described solely by name; describe more complex techniques in the Methods section.*
☒	☐	A description of all covariates tested
☐	☒	A description of any assumptions or corrections, such as tests of normality and adjustment for multiple comparisons
☐	☒	A full description of the statistical parameters including central tendency (e.g. means) or other basic estimates (e.g. regression coefficient) AND variation (e.g. standard deviation) or associated estimates of uncertainty (e.g. confidence intervals)
☐	☒	For null hypothesis testing, the test statistic (e.g. F, t, r) with confidence intervals, effect sizes, degrees of freedom and P value noted<br>*Give P values as exact values whenever suitable.*
☒	☐	For Bayesian analysis, information on the choice of priors and Markov chain Monte Carlo settings
☒	☐	For hierarchical and complex designs, identification of the appropriate level for tests and full reporting of outcomes
☒	☐	Estimates of effect sizes (e.g. Cohen's *d*, Pearson's *r*), indicating how they were calculated

Our web collection on statistics for biologists contains articles on many of the points above.

Software and code

Policy information about availability of computer code

Data collection	SerialEM v4.0
Data analysis	AxioVision Se64, Graphpad Prism, Image Studio Lit, ImageJ, SnapGene, ZenBlue; cryoSPARC v3.6.0, FREALIGN v9.11, RELION v5.0, deepEMhancer v0.1, Chimera v1.17, ChimeraX v1.8, AlphaFold2, Alphafold3, Coot v0.9.8, Phenix v1.20, ModelAngelo v0.3, DeepTracer (no version number), DeepTracerID (no version number), DomainSeeker (no version number), DomainSeeker v1.11, SITUS v3.1, Mascot v2.7.0, custom scripts available from https://github.com/rui--zhang/Microtubule and https://github.com/rui--zhang/Doublet

For manuscripts utilizing custom algorithms or software that are central to the research but not yet described in published literature, software must be made available to editors and reviewers. We strongly encourage code deposition in a community repository (e.g. GitHub). See the Nature Portfolio guidelines for submitting code & software for further information.

Data

Policy information about availability of data

All manuscripts must include a data availability statement. This statement should provide the following information, where applicable:

- Accession codes, unique identifiers, or web links for publicly available datasets
- A description of any restrictions on data availability
- For clinical datasets or third party data, please ensure that the statement adheres to our policy

Cryo-EM structures have been deposited to the Electron Microscopy Data Bank with accession codes EMD-72715 (conoid fiber, 24-nm repeat length), EMD-72716

(PCR-P2), EMD-72717 (ICMT-1), EMD-72718 (ICMT-2) and EMD-72719 (apical SPMT). Corresponding atomic models have been deposited in the Protein Data Bank with accession codes 9Y9Z, 9YA0, 9YA1, 9YA2 and 9YA3, respectively. The mass spectrometry proteomics data have been deposition to the ProteomeXchange Consortium via the PRIDE partner repository with the dataset identifier PXD068413 and 10.6019/PXD068413.

Research involving human participants, their data, or biological material

Policy information about studies with human participants or human data. See also policy information about sex, gender (identity/presentation), and sexual orientation and race, ethnicity and racism.

Reporting on sex and gender	n/a
Reporting on race, ethnicity, or other socially relevant groupings	n/a
Population characteristics	n/a
Recruitment	n/a
Ethics oversight	n/a

Note that full information on the approval of the study protocol must also be provided in the manuscript.

Field-specific reporting

Please select the one below that is the best fit for your research. If you are not sure, read the appropriate sections before making your selection.

☒ Life sciences ☐ Behavioural & social sciences ☐ Ecological, evolutionary & environmental sciences

For a reference copy of the document with all sections, see nature.com/documents/nr-reporting-summary-flat.pdf

Life sciences study design

All studies must disclose on these points even when the disclosure is negative.

Sample size	The number of data points collected from each sample was based on the minimum required to perform statistical comparisons (n≥3). All in vitro experiments were performed at least two independent times. No statistical methods were used to predetermine sample size. For cryo-EM processing, no methods were used to predetermine sample size. The size of the cryo-EM datasets was determined by the need to identify proteins and build an atomic model. The number of micrographs and particles are listed in the Extended Data.
Data exclusions	Micrographs with low resolution estimates following CTF fitting were discarded. The algorithms used for image processing may down-weigh or exclude particles as part of their refinement strategy. No other data points were excluded for the analysis.
Replication	All in vitro experiments were repeated at least 2 or 3 times independently with 2-3 technical replicates for each. All in vitro results were successfully replicated. Two replicates of the treated T. gondii sample were subject to independent mass spectrometry analysis, which yielded similar results. Cryo-EM maps represent an average of many thousands of individual copies of the complex of interest, collected from multiple preparations across several microscope sessions.
Randomization	For calculation of the Fourier Shell Correlation (FSC), cryo-EM particles were randomly split into two halves.
Blinding	Blinding is not necessary since there are no groups that need subjective analysis.

Reporting for specific materials, systems and methods

We require information from authors about some types of materials, experimental systems and methods used in many studies. Here, indicate whether each material, system or method listed is relevant to your study. If you are not sure if a list item applies to your research, read the appropriate section before selecting a response.

Materials & experimental systems

n/a	Involved in the study	
☐	☒	Antibodies
☐	☒	Eukaryotic cell lines
☒	☐	Palaeontology and archaeology
☒	☐	Animals and other organisms
☒	☐	Clinical data
☒	☐	Dual use research of concern
☒	☐	Plants

Methods

n/a	Involved in the study	
☒	☐	ChIP-seq
☒	☐	Flow cytometry
☒	☐	MRI-based neuroimaging

Antibodies

Antibodies used

Antibodies for immunofluorescence assay (IFA) include Mouse anti-Ty, Mouse anti-HA.11 (BioLegend, Cat#901501), rabbit anti-HA (BioLegend, Cat#71-5500), Rat anti-HA (Millipore Sigma, Cat#11867423001), Mouse anti-Myc (BioLegend, Cat#626802); Chicken anti-Myc (Thermo Fisher, Cat#A21281); Mouse anti-AceTub (Sigma, Cat#T7451), Rabbit anti-AceTub (Cell Signalling Technology, Cat#5335T) and Rabbit anti-TgAldolase (in-house).
For secondary antibodies, Alexa Fluor 488 Goat anti-mouse IgG (H+L) (Thermo Fisher, Cat#A-11029); Alexa Fluor 488 Goat anti-rabbit IgG (H+L) (Thermo Fisher, Cat#A-11008);Alexa Fluor 568 Goat anti-mouse IgG (H+L) (Thermo Fisher, Cat#A-11031); Alexa Fluor 568 Goat anti-rabbit IgG (H+L) (Thermo Fisher, Cat#A-11011); Goat anti-Chicken, adsorbed, DyLight 350 (Thermo Fisher, Cat#SA5-10069); IRDye 800CW Goat anti-mouse IgG (H+L) (LI-COR Biosciences, Cat#925-32210);IRDye 800CW Goat anti-rabbit IgG (H+L) (LI-COR Biosciences, Cat#925-32211); IRDye 680RD Goat anti-mouse IgG (H+L) (LI-COR Biosciences, Cat#925-68070);IRDye 680CW Goat anti-rabbit IgG (H+L) (LI-COR Biosciences, Cat#925-68071)

Validation

Mouse anti-HA, rabbit anti-HA antibody, Rat anti-HA, Mouse anti-Myc, Chicken anti-Myc, Mouse anti-AceTub, Rabbit anti-AceTub were validated by the manufacturers as described in the product description.
Mouse anti-Ty: in house hybridoma was originally obtained from: Bastin, P., Bagherzadeh, A., Matthews, K. R. & Gull, K. A novel epitope tag system to study protein targeting and organelle biogenesis in Trypanosoma brucei. Mol. Biochem. Parasitol. 77, (1996). It was validated in the lab by testing against protein standards bearing this epitope tag.
Rabbit anti-TgAldolase: Starnes GL, Jewett TJ, Carruthers VB, Sibley LD (2006) Two separate, conserved acidic amino acid domains within the Toxoplasma gondii MIC2 cytoplasmic tail are required for parasite survival. J Biol Chem 281:30745–30754
Mouse anti-TgSAG1: Burg J.L. Perelman D. Kasper L.H. Ware P.L. Boothroyd J.C. Molecular analysis of the gene encoding the major surface antigen of Toxoplasma gondii. J. Immunol. 1988; 141: 3584-3591.

Eukaryotic cell lines

Policy information about cell lines and Sex and Gender in Research

Cell line source(s)

Human Foreskin Fibroblasts (HFFs, ATCC, Cata#CRL-1634); all T. gondii lines were obtained from published studies or generated by this study;

Authentication

All T. gondii strains were validated by PCR and sequencing;HFFs from ATCC were not authenticated.

Mycoplasma contamination

They are checked regularly using an e-MycoPlasma kit as stated in the methods.

Commonly misidentified lines
(See ICLAC register)

No commonly misidentified cell lines were used in the study.

Plants

Seed stocks

n/a

Novel plant genotypes

n/a

Authentication

n/a
