# Supplementary material for: Atomic models of the Toxoplasma cell invasion machinery
Source: Nat Struct Mol Biol. 2025 Dec 9;33(1):157–70. doi: 10.1038/s41594-025-01728-w (PMC12819142; doi:10.1038/s41594-025-01728-w)

Source data Fig. 7d
Western blotting of FRM1-mAID-HA

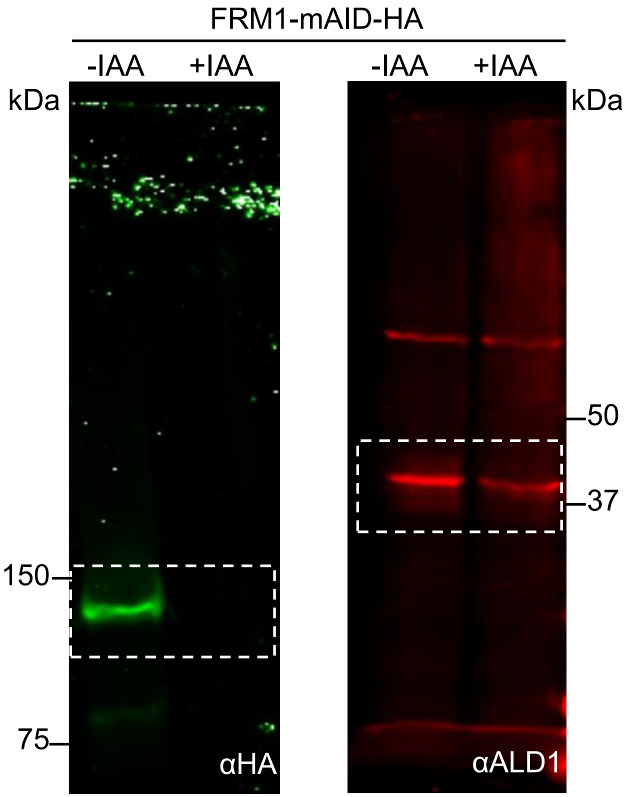

Source data Fig. 7d
Western blotting of FLM1-mAID-HA

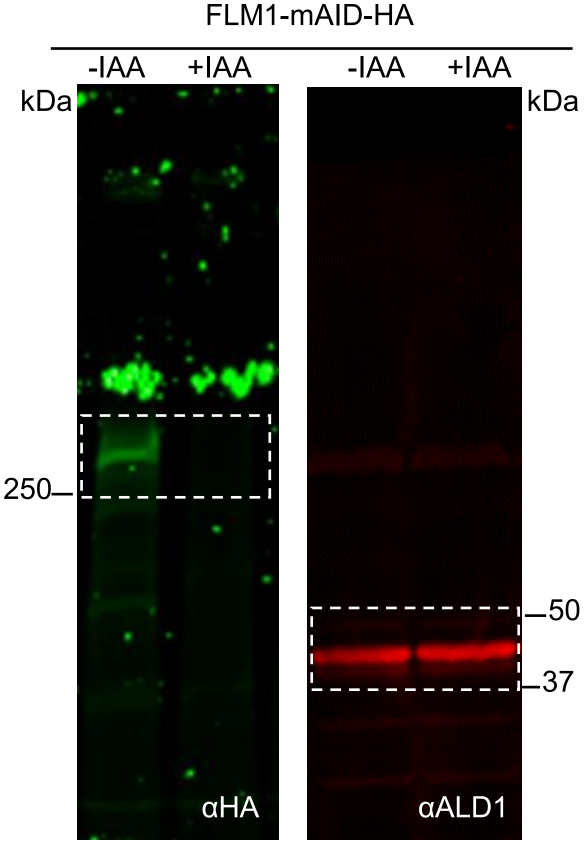

Source data Fig. 7d
Western blotting of FLM2-mAID-HA

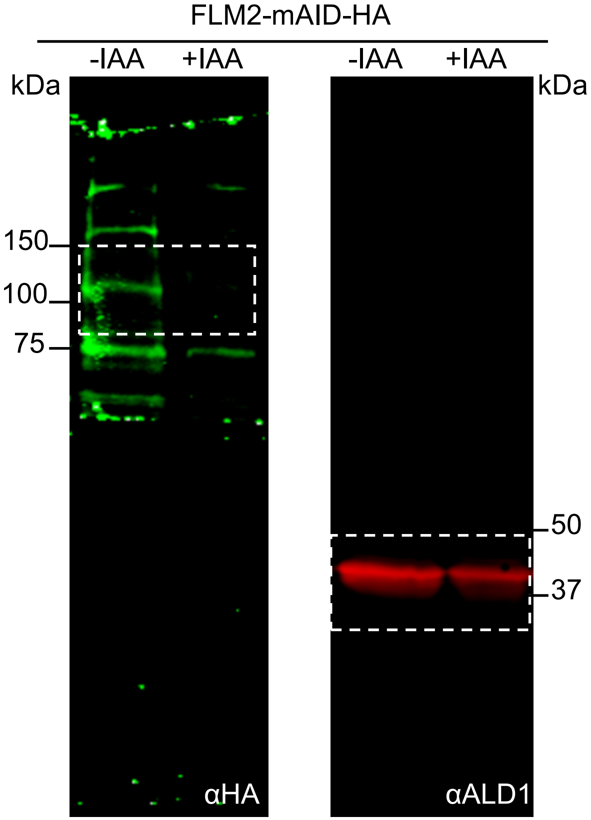

Source data Fig. 7d
Western blotting of AKMT2-mAID-HA

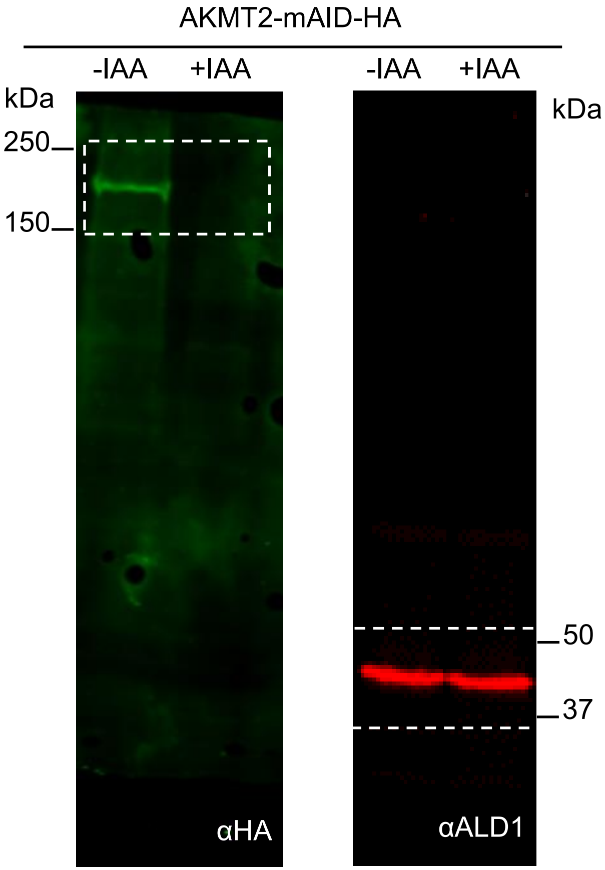

Source data Fig. 7d
Western blotting of MyoL-mAID-HA

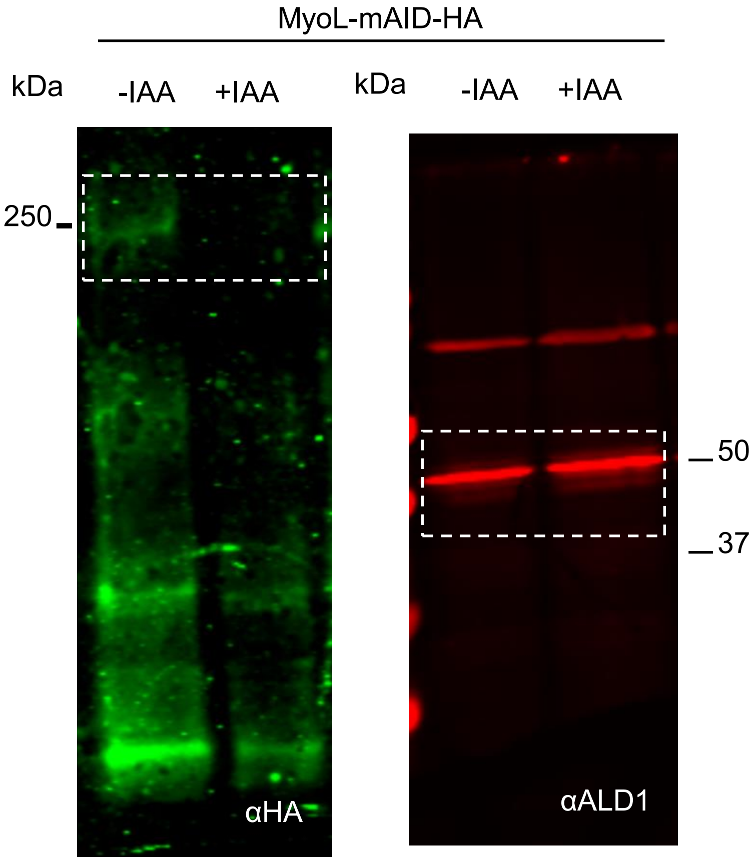

Source data Fig. 7d
Western blotting of CAM4-mAID-HA

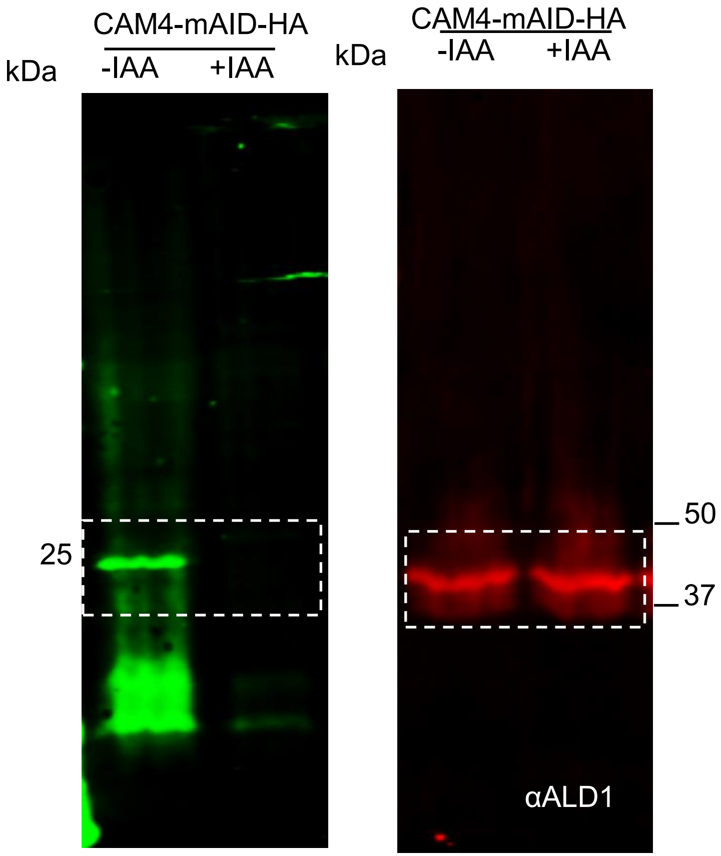

Source data Fig. 7d
Western blotting of SEC24-mAID-HA

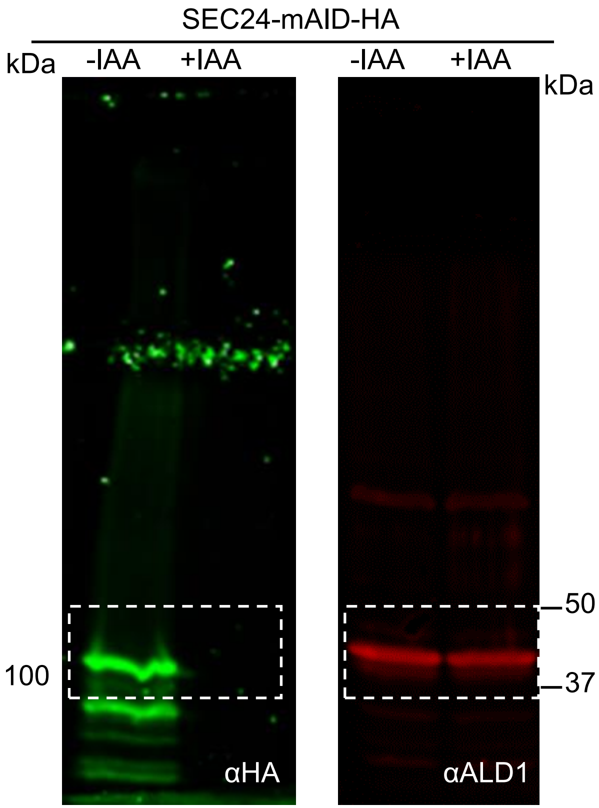

Supplement: Supplementary file 7 — Original western blot images. [file 41594_2025_1728_MOESM7_ESM.pdf]
