# Supplementary material for: Atomic models of the Toxoplasma cell invasion machinery
Source: Nat Struct Mol Biol. 2025 Dec 9;33(1):157–70. doi: 10.1038/s41594-025-01728-w (PMC12819142; doi:10.1038/s41594-025-01728-w)
Supplement: Supplementary file 9 — Original western blot images. [file 41594_2025_1728_MOESM9_ESM.pdf]

# Source Data Extended Data Fig. 4b

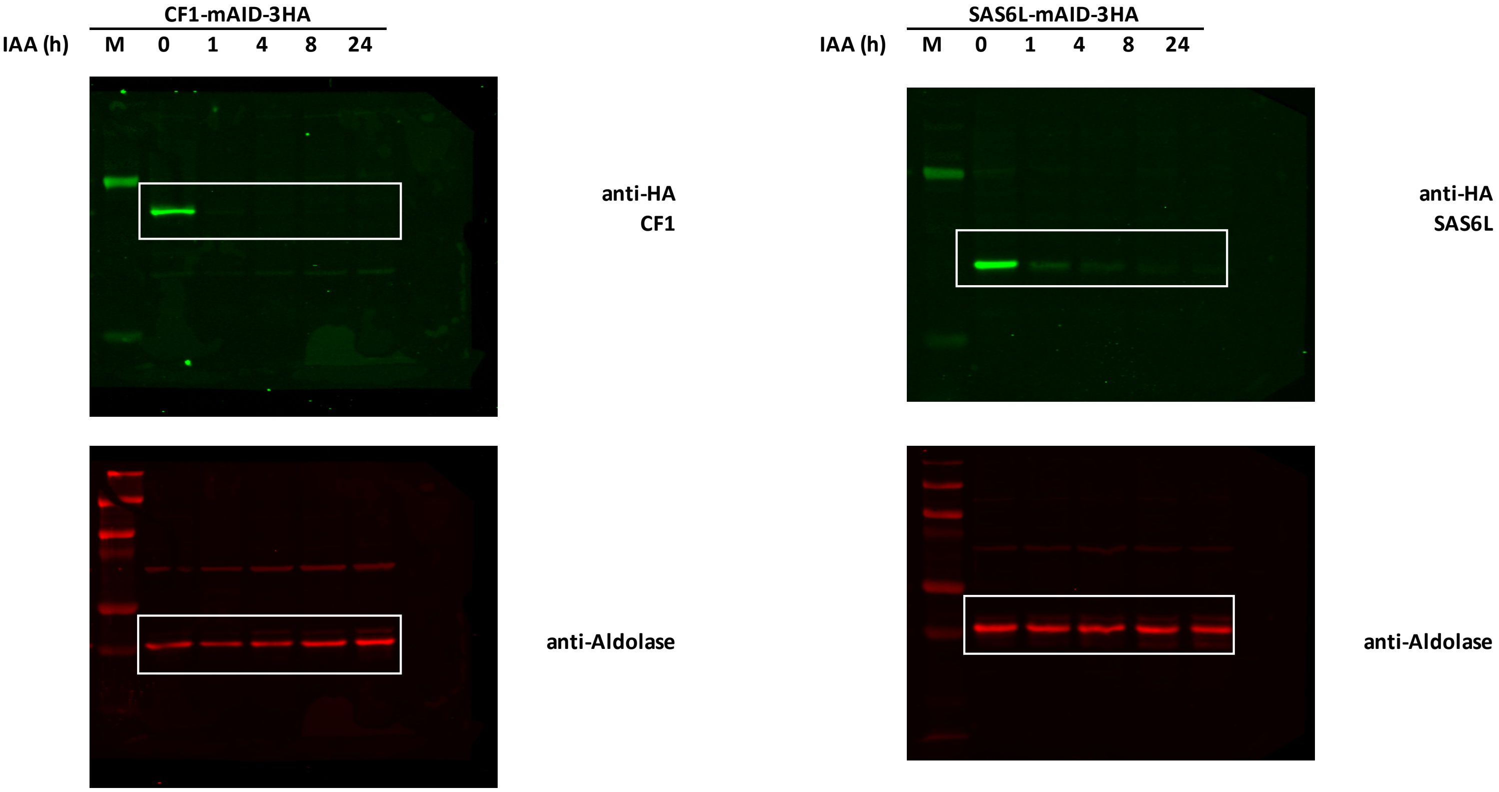

# Source Data Extended Data Fig. 4d

SAS6L-mAID-3HA

IAA - + - + - +

M CF1 CF5 CIP3

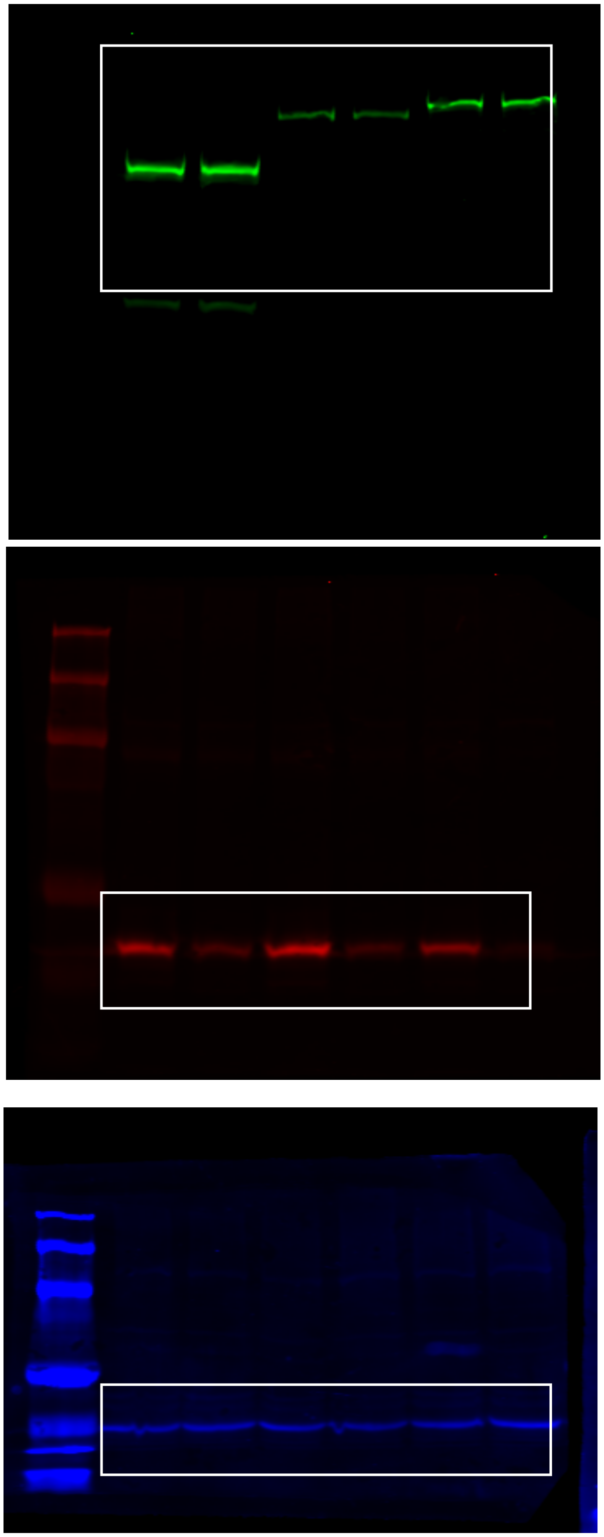

anti-Myc
CF proteins

anti-HA
SAS6L

anti-Aldolase
