# Supplementary material for: Atomic models of the Toxoplasma cell invasion machinery
Source: Nat Struct Mol Biol. 2025 Dec 9;33(1):157–70. doi: 10.1038/s41594-025-01728-w (PMC12819142; doi:10.1038/s41594-025-01728-w)

Source Data Extended Data Fig. 9b
Western blotting assay for PCR10-mAID-HA parasites

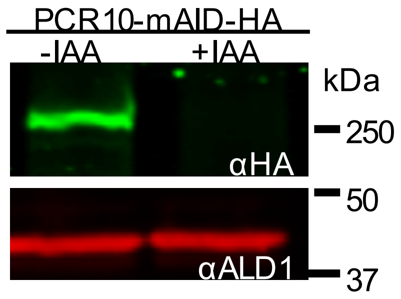

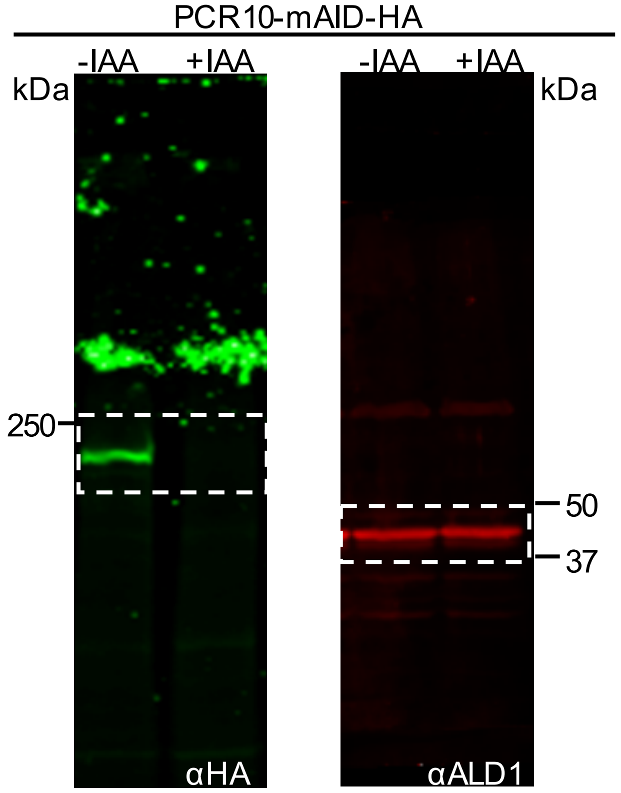

Source Data Extended Data Fig. 9d
Western blotting assay for SEC23-mAID-HA parasites

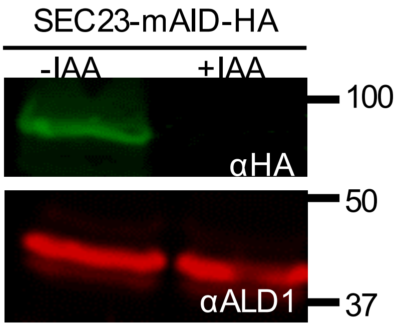

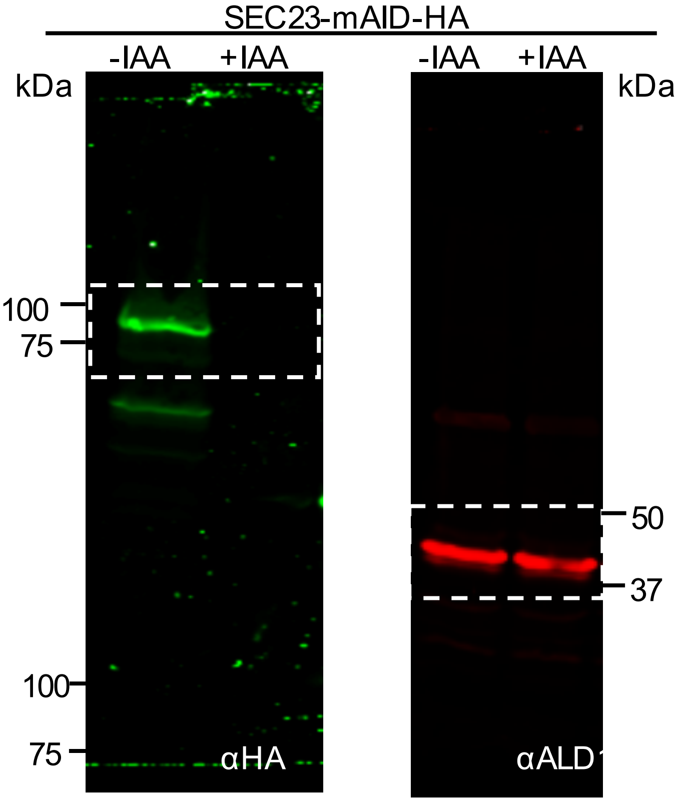

Supplement: Supplementary file 11 — Original western blot images. [file 41594_2025_1728_MOESM11_ESM.pdf]
